# Women and Whisky

**Published:** 1904-07-16

**Authors:** 


### EDITOR'S LETTER-BOX.

[Our Correspondents are reminded that prolixity is a great bar to publication, and that brevity of style and conciseness of statement greatly facilitate early insertion.]

### WOMEN AND WHISKY.

*To the Editor of* THE HOSPITAL.

"AN ONLOOKER" writes: Having been travelling I have only just seen THE HOSPITAL for June 4th, and a short article there has attracted my attention. You refer to a lady who in the *Graphic* has accused doctors of unjustifiably recommending whisky to women, "and so bringing them to their ruin." In protesting against this accusation you say (the italics are mine) the physician advises the *restricted* employment of a stimulant or drug; he is not to blame if his patient subsequently uses it without restriction. Adults of *sane* mind are accountable for their own actions. On these two words the whole thing turns. If doctors always prescribed the exact dose of stimulant to be taken, and the *length of time during which it should be continued*, they would indeed have no responsibility in ordinary cases. But what they often do is to say, "Take a little port," or, worst of all, "Have a glass of hot whisky and water before you go to bed. That will make you sleep." I know at this moment a woman —a domestic servant—whose father was a drunkard. She gets bottles of cheap port wine from the grocer, as I happened to have discovered. She says the doctor ordered it. When I ask how long ago she says, "Oh, last year." When I ask "Didn't he say how long you were to go on?" she says, "No, he just said, 'Take a little port.'" And I believe it, because

I have heard doctors do it again and again. And this case brings me to the next point. Although never for a moment wishing to shift moral responsibility on to the wrong shoulders, the question may well be asked, "Where does the *sane* mind begin? Doctors will order stimulant without any inquiry into the family history of the patient. I know a lady whose family history has been of the worst. The doctors have for years ordered her whisky for internal pain. She is now an inebriate. It is futile to ask whether the patient, the heredity, or the doctor is responsible. Let him, at all events, do his best to avoid so terrible a possibility. Sir, I have long desired that someone should appeal to doctors in this matter. The profession is full of men who have the real welfare of their fellows at heart. I am sure they will not pass the matter by.

